# A brief early intervention for adolescent depression that targets emotional mental images and memories: protocol for a feasibility randomised controlled trial (IMAGINE trial)

**DOI:** 10.1186/s40814-018-0287-3

**Published:** 2018-07-04

**Authors:** Victoria Pile, Patrick Smith, Mary Leamy, Simon E. Blackwell, Richard Meiser-Stedman, Dominic Stringer, Elizabeth G. Ryan, Barnaby D. Dunn, Emily A. Holmes, Jennifer Y. F. Lau

**Affiliations:** 1 Department of Psychology, Institute of Psychiatry, Psychology and Neuroscience, King’s College London, De Crespigny Park, London, SE5 8AF UK; 2 Florence Nightingale Faculty of Nursing and Midwifery, King’s College London, London, UK; 3 Mental Health Research and Treatment Center, Department of Psychology, Ruhr-Universität Bochum, Bochum, Germany; 4 Department of Clinical Psychology, University of East Anglia, Norwich, UK; 5 Mood Disorders Centre, University of Exeter, Exeter, UK; 6 Department for Clinical Neuroscience, Karolinska Institutet, Stockholm, Sweden; 7 Department of Biostatistics and Health Informatics, Institute of Psychiatry, Psychology and Neuroscience, King’s College London, London, UK

**Keywords:** Depression, Adolescence, Mental imagery, Autobiographical memory, Early intervention, Psychological therapy

## Abstract

**Background:**

Adolescent depression is common and impairing. There is an urgent need to develop early interventions to prevent depression becoming entrenched. However, current psychological interventions are difficult to access and show limited evidence of effectiveness. Schools offer a promising setting to enhance access to interventions, including reducing common barriers such as time away from education. Distressing negative mental images and a deficit in positive future images, alongside overgeneral autobiographical memories, have been implicated in depression across the lifespan, and interventions targeting them in adults have shown promise. Here, we combine techniques targeting these cognitive processes into a novel, brief psychological intervention for adolescent depression. This feasibility randomised controlled trial will test the feasibility and acceptability of delivering this imagery-based cognitive behavioural intervention in schools.

**Methods/design:**

Fifty-six adolescents (aged 16–18) with high symptoms of depression will be recruited from schools. Participants will be randomly allocated to the imagery-based cognitive behavioural intervention (ICBI) or the control intervention, non-directive supportive therapy (NDST). Data on feasibility and acceptability will be recorded throughout, including data on recruitment, retention and adherence rates as well as adverse events. In addition, symptom assessment will take place pre-intervention, post-intervention and at 3-month follow-up. Primarily, the trial aims to establish whether it is feasible and acceptable to carry out this project in a school setting. Secondary objectives include collecting data on clinical measures, including depression and anxiety, and measures of the mechanisms proposed to be targeted by the intervention. The acceptability of using technology in assessment and treatment will also be evaluated.

**Discussion:**

Feasibility, acceptability and symptom data for this brief intervention will inform whether an efficacy randomised controlled trial is warranted and aid planning of this trial. If this intervention is shown in a subsequent definitive trial to be safe, clinically effective and cost-effective, it has potential to be rolled out as an intervention and so would significantly extend the range of therapies available for adolescent depression. This psychological intervention draws on cognitive mechanism research suggesting a powerful relationship between emotion and memory and uses imagery as a cognitive target in an attempt to improve interventions for adolescent depression.

**Trial registration:**

ISRCTN85369879

## Background

Depression is common and costly, with adolescence being a crucial period for its onset and long-term impact [1]. The peak age of first onset of depression is in adolescence, with onset during adolescence (rather than adulthood) associated with more recurrences and an increased risk of chronicity [2, 3]. Adolescent depression impacts on functioning, being associated with higher social dysfunction, poorer academic performance, more physical ill health complaints and more completed suicides [4–6]. Intervention during the early stages of depression may prevent the long-lasting and severe outcomes associated with it (as well as prevent the development of secondary disorders) [3]. Yet, current early interventions are difficult to access [7] and lack convincing evidence of effectiveness [8]. Access could be improved by delivering interventions in school settings to reduce barriers of time and cost [8–10], and targeting the underlying mechanisms that drive depression could reduce risk for future relapse. Two promising new treatment targets are mental imagery and autobiographical memory specificity. These targets are suggested from evidence that people with depression frequently experience past distressing images, struggle to imagine positive events in their future and do not recall memories in detail [11–15]. Adolescence is a key period to target these processes, given that depressive symptoms commonly begin in adolescence, cognitive factors are likely to stabilise during this time and adolescents may harness imagery techniques more readily than verbal approaches.

We have developed a novel and brief intervention that targets both mental imagery and autobiographical memory through a series of exercises. The simple and manualised format has a potential for future scalability, since it may not require an expert therapist to administer. Here, we aim to investigate whether such an early intervention is feasible and acceptable in a feasibility randomised controlled trial (RCT). We will also gather descriptive data on changes in symptoms and the proposed mechanisms, as well as evaluate the role that technology could play in enhancing assessment and intervention for this population.

### Mental imagery

Mental images range from distressing memories of past events, entering awareness unbidden and producing strong psychophysiological stress responses (i.e. intrusions) to images of possible future events deliberately brought to mind (e.g. when planning) [11]. Mental imagery can be positive or negative and, unlike verbal thoughts, is characterised by having close access to sensory information, being as if ‘seeing with the mind’s eye or hearing with the mind’s ear’ [16]. In adults, depression is associated with both an increased frequency of distressing intrusive images and a deficit in generating positive future imagery [13, 15, 17–20]. Whilst these associations have mostly been demonstrated in adults, there is increasing evidence also documenting these relationships in adolescence [14]. A number of techniques have been explored to target mental imagery, including imagery rescripting to process distressing negative images and techniques to enhance positive imagery. Both techniques have demonstrated promising results in depression and other disorders in adults [21–26]. The current intervention aims to transform negative images and challenge the meaning associated with a non-traumatic negative life event as well as enhance the vividness and positive emotional response to positive images.

Targeting imagery in adolescence could be particularly developmentally appropriate. Compared to adults, adolescents may rely more generally on imagery for processing and skill acquisition [27]. Moreover, in response to extreme stressors, factors that change during development (such as encoding, emotional regulation, memory retrieval) may mean that young people are less able to cognitively process stressful experiences compared to adults [14, 28, 29], perhaps putting them at higher risk for developing intrusive images. Studies have demonstrated that non-traumatic stressful life events in youth (such as bullying) can have long-term adverse effects on mental health and these events can enter awareness as distressing negative images [14, 30]. Indeed, distressing recurrent mental images linked to disorder onset frequently begin in adolescence [31]. Therefore, imagery interventions could be particularly suited to this period of cognitive development, with adolescence representing a balance between heightened reliance on image-based processing (relative to adults) and greater degree of cognitive control over emotional mental imagery (relative to children). More generally, adolescence represents a period of increased flexibility and learning potential. This may mean negative experiences, such as depression, alter developmental trajectories resulting in long-lasting maladaptive changes that persist into adulthood [32–35]. Yet, due to this greater plasticity, adolescence could also be a key period of opportunity to prevent chronic depression developing.

### Memory specificity

Autobiographical memory relates to the ability to recollect facts and personal events from one’s life [36]. The specificity of this recollection is variable across people. Specific memories identify unique events, occurring at a particular time and place, and are in contrast with memories that are of repeated events (categorical memories) or events that last longer than a day (extended memories) [19]. Overgeneral memory (OGM) is a phenomenon where individuals have difficulty retrieving specific autobiographical memories and instead generate categorical or extended memories. Autobiographical memory is important for the individual’s sense of self and their daily functioning. Difficulties in accessing and processing specific autobiographical memory impact on daily cognitive functioning, including planning, problem-solving and social interaction [20] as well as difficulties imagining future events [37].

OGM has been consistently implicated in adolescent depression, being not only associated with current symptoms but also with the onset, maintenance and relapse of depression [38]. Research in adults has further demonstrated that OGM is predictive of developing depression [39] and of later depression severity, even when one is not currently depressed [40]. Researchers suggest that people may try to avoid the details of a negative event in order to control their response to it and that this reduces specificity across positive and negative memories [19]. For example, the experience of childhood abuse is associated with OGM in young people [41], and OGM predicts depression severity following trauma in adults [42]. Importantly, research with adults has demonstrated that OGM does not reflect a general deficit in memory functioning [19] and is not purely a correlate of current low mood [43]. Early-stage trials in adolescents and adults indicate that a simple approach to increase memory specificity (generating specific memories to cue words, e.g. happy) can reduce depressive symptomatology and improve day-to-day cognition [24, 44, 45].

### Aims of IMAGINE trial

Here, we have combined techniques of imagery rescripting for negative events, image generation for positive events and memory specificity training to produce a novel early intervention for adolescent depression. The intervention is brief, manualised and clearly structured which will facilitate future scalability through delivery by practitioners without extensive training. The primary aim of the IMAGINE (Integrating Memories And Generating Images of New Experiences) trial is to investigate whether it is feasible and acceptable to carry out a RCT of this novel intervention (compared to a suitable control condition) successfully within a school setting. Given that a future trial would need to assess whether the proposed active components of the imagery-based cognitive behavioural intervention are contributing to any change (rather than other non-specific therapist factors, such as empathy and active listening), the comparison intervention will include non-specific therapist factors. The secondary aim is to collect pilot data on clinical measures as well as on the underlying mechanisms proposed to be targeted in the intervention. This will include measures of imagery vividness, emotional response to a positive memory and memory specificity. Finally, the trial will also assess the feasibility and acceptability of incorporating technology into assessment and the intervention.

## Methods/design

### Study design and timeline

**Fig. 1:**
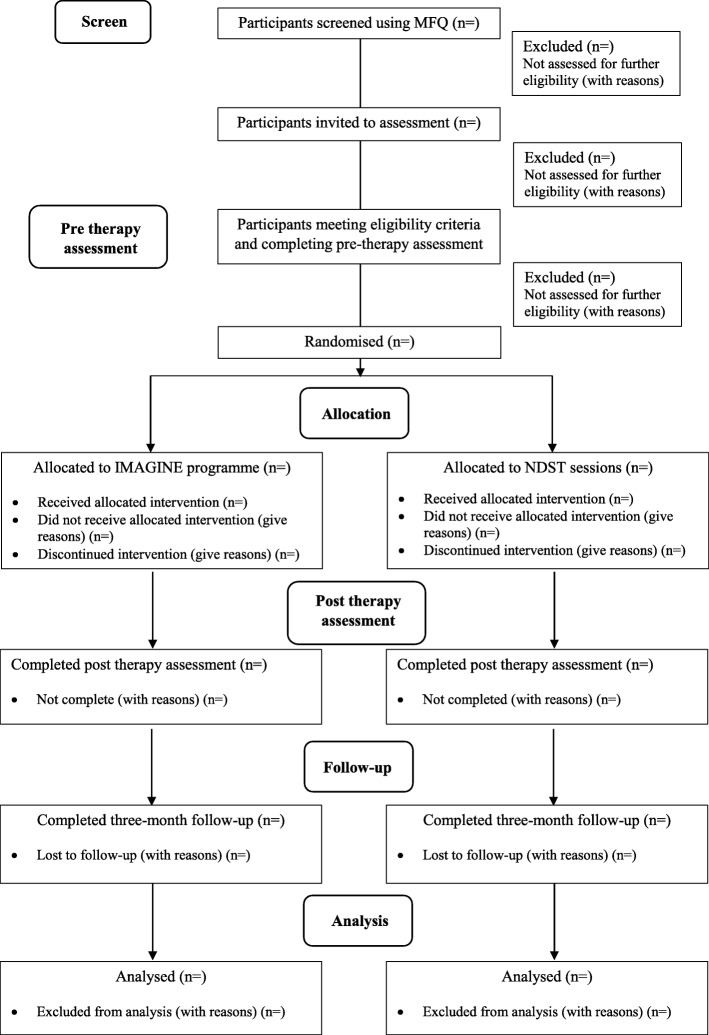
IMAGINE trial flowchart

**Fig. 2:**
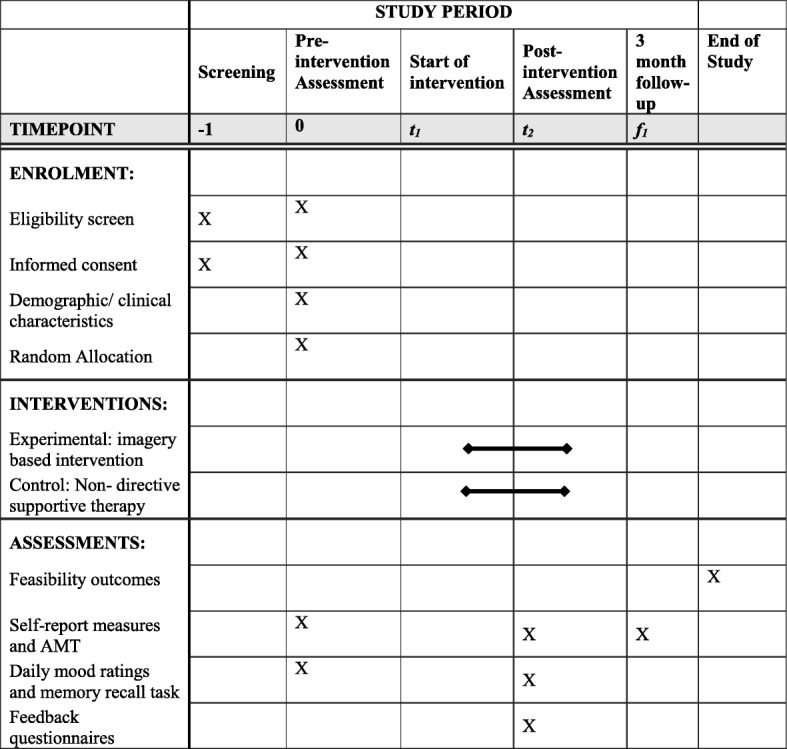
SPIRIT figure for IMAGINE trial

This study is a feasibility randomised controlled trial with parallel groups, conducted across multiple schools in the United Kingdom (UK). Both groups will receive active interventions, and both interventions aim to improve mood and self-esteem. Participants will be assessed at pre-intervention (prior to randomisation), and follow-up assessments will take place after the intervention and a briefer assessment completed 3 months following the end of the intervention (see Figs. 1 and 2). These methods are based on the IMAGINE trial protocol (version 1; 1 April 2017), approved by the trial steering committee.

### Study population

#### Inclusion criteria

1. Aged 16–18 (a narrow age range was chosen to reduce heterogeneity between the groups, for example to reduce the influence of individual differences in maturational and experiential factors).
2. Informed consent (obtained by the assessor during the pre-intervention assessment).
3. Willing and able to engage in psychological intervention and complete assessments.
4. Scoring above 20 on the Mood and Feelings Questionnaire (MFQ of 33 items) in the pre-intervention assessment. The MFQ has 70% sensitivity and 81% specificity for discriminating any mood disorder [46]. A score of 20 is based on similar previous studies [47] and trial protocols [48].

#### Exclusion criteria

1. Diagnosis of learning disability or significant head injury, neurological disorder or epilepsy.
2. Unable to fluently communicate in English.
3. Unable to give informed consent.
4. Factors contra-indicating imagery rescripting, i.e. high levels of current risk. This will be verbally assessed with the participant in the pre-intervention assessment.
5. Currently receiving another psychological intervention (including school counselling).
6. Experiencing distressing psychotic symptoms or depressed in the postnatal period (participants with comorbid physical illness or non-psychotic disorders, such as anxiety, will not be excluded).

#### Sample size

A power calculation to determine the sample size is not appropriate for a feasibility trial, as the purpose of the trial is not to establish efficacy at this stage. However, the data from this trial could be used to provide initial estimates of the likely range of effect sizes for the intervention. The target recruitment for this feasibility trial will be *N* = 56 (28 in each arm). This was determined with reference to good practice recommendations for feasibility studies, which recommend sample sizes of between 24 and 50 [49–51]. The sample size of 50 was inflated to allow for drop-out following randomisation, which was estimated to be 10% based on similar studies [47] identifying a final sample size of 56.

#### Recruitment, randomisation and blinding

All participants will be recruited from secondary schools and sixth form colleges in the UK. For the screening stage, information sheets will be provided and young people asked to complete the Mood and Feelings Questionnaire (MFQ, at screening the four risk items will be removed from the MFQ due to ethical considerations). Eligible participants will be contacted and provided with the information sheet for the intervention stage. Young people will be given at least 24 h to read over the information and consider taking part, as well as discuss their participation with anyone they may wish to (e.g. parent). The first assessment will then be completed at the young person’s school, which will include administering the MFQ again (33-item version) and confirming the other eligibility criteria. Once the young person has given informed consent, the full pre-intervention assessment will be administered. Whilst parental consent will not be sought, since all participants will be over age 16, we will follow each school’s recommendations for contacting parents and discussing participation. Examples of this might be contacting the parents by phone, email or letter and providing information about the trial and the opportunity to contact the team and discuss the study. A number of strategies attempting to enhance recruitment and retention have been incorporated; these include recommendations from service user consultants to deliver the intervention in schools, to ask young people about the best way to contact them to discuss the project and to not complete formal diagnostic assessments. Furthermore, specific strategies will be discussed with individual schools as to how best to recruit young people and retain them in the trial.

Following the assessment, participants will be randomly allocated to one of two interventions by the King’s Clinical Trials Unit (KCTU). Participants will be randomised in a 1:1 ratio using block randomisation via a web-based system. The sample will be stratified by school. Randomly varying block sizes will reduce the predictability of the sequence and ensure allocation concealment. The control intervention is a recommended Tier 2 intervention, which helps to address potential ethical issues related to randomisation. The randomisation system will be accessed by the chief investigator (VP) via the web interface in the time period between the pre-intervention assessment and first intervention session.

A double-blind design is not possible due to the nature of the intervention under investigation (the young person will be aware of which intervention they have received, as will the therapist administering it). However, as both experimental and control interventions are credible therapeutic interventions, this should help reduce any potential bias associated with expectations of the benefits of the intervention. The two interventions will be referred to as intervention 1 and intervention 2, and both will be described as ‘programmes aiming to improve low mood and self-esteem’ in all participant and staff literature in order to promote equal intervention credibility between the conditions. That is, participants are not informed as to what is the ‘new’ intervention in order to avoid potential imbalances in expectancy. All reasonable attempts will be made to keep school staff blind as to which intervention participants have been randomised to.

VP will be primarily responsible for gathering the data and will conduct both therapeutic interventions. Assessments will be completed by appropriately trained individuals, independent from the clinical team (e.g. research assistants and postgraduate students), who will be blind to intervention allocation. Participants who drop out or discontinue the intervention will still be invited to complete the assessment sessions. For clinical data collection, risk of assessor bias will also be reduced by choosing measures that are less susceptible to bias and by using multiple measures. Good Clinical Practice guidelines for clinical trials will be followed [52].

### Interventions

Both interventions will consist of three to four individual sessions with VP. Four sessions will be offered to young people taking part in the trial, and sessions will be re-arranged where possible. Successful completion of the intervention is defined as not missing more than one intervention session (i.e. completing three sessions). It is anticipated that the most likely reason for young people attending three rather than four sessions is that they miss one session and then there not being sufficient time remaining in the school term to re-arrange.

#### Experimental intervention: imagery-based cognitive behavioural intervention

The intervention will follow a written treatment manual and is accompanied by a workbook. The intervention will combine (A) imagery rescripting to reduce the distress associated with certain negative images and build positive future images and (B) memory specificity training to increase specificity and access to memories. The intervention is based on cognitive behavioural principles and experimental psychopathology research on mental imagery and emotion. An overview of the intervention is outlined below:

1. Rationale for ‘training memories’, including the role of memories and relationship between memories/images, mood and behaviour.
2. Imagery rescripting for a negative past image (e.g. a negative experience in school). The procedure will be based on previous adult literature where participants visit the memory in three stages [23, 53, 54]. Unlike the adult literature, this intervention is not aiming to rescript trauma memories. Participants will be asked to choose an image that is negative that they have thought about recently and that is associated with school (for example failing an exam or an argument with friends).
3. Scripting positive future imagery (e.g. graduating from university) using a similar protocol as above, adapted for future goals.
4. Rationale and practice for making memories more specific and detailed. Specificty training will include in-session exercises and homework tasks delivered using daily prompts (e.g. participants will be asked to generate a memory to a cue word).

#### Control intervention

A structured control intervention, based on previous studies [55–57], will be delivered. This will consist of ‘non-directive supportive therapy’ (NDST) as this is the NICE recommended treatment for depression in Tier 2 [57]. NDST involves the planned delivery of individual sessions with an empathic, concerned professional for emotional support, discussion of participant-initiated options for addressing problems and monitoring (e.g. depressive symptoms). Participants in the NDST arm will be offered the same number and frequency of sessions as those in the imagery-based cognitive behavioural intervention (ICBI) arm. This intervention is designed to control for factors that, other than active components of therapy, could contribute to change, such as passage of time and non-specific aspects of therapy (e.g. speaking to an empathic therapist). The intervention will follow a written treatment manual.

#### Treatment fidelity

Sessions will be audio recorded (if the young person consents). A sample of these sessions will then be assessed for adherence to the intervention manuals by an independent rater. The rater will assess the audio recordings for both adherence and competency, according to a modified version of the cognitive therapy scale [58]. Ratings of adherence to the manuals will also indicate whether there has been contamination between the conditions from the therapist having knowledge of both interventions.

### Outcome measures

#### Primary objective—feasibility/acceptability data (see Fig. 1)

##### Number of eligible participants, willingness of schools and participants to take part and be randomised

1. Recruitment throughout trial.
  Number of schools that are approached and number of schools agreeing to take part (as well as proportion of total schools that are approached).
  Number of young people potentially eligible to complete screening questionnaire.
  Number (and proportion of relevant total) of participants that complete the screening stage.
  Number of eligible participants at screening and at pre-intervention assessment as well as proportion of total.
  Number (and proportion) of ineligible participants at screening and at pre-intervention assessment (and reasons for this).
  Number (and proportion) of participants consented to take part in the intervention.
  Number (and proportion) of participants randomised.
2. Number of participants successfully completing intervention and reasons for non-completion (successful completion is defined as not missing more than one intervention session).
  Number (and proportion) of participants who drop out and reasons for this. (In line with our ethical informed consent procedure, participants can discontinue or withdraw from the study at any time without giving a reason. Reasons for discontinuation/withdrawal will be recorded when possible.)

##### Follow-up rates, response rates to questionnaires, adherence/compliance rates

1. Data on retention at each time point (pre-intervention, post-intervention and 3-month follow-up assessments).
2. During the intervention: range and average number of sessions completed (including number of sessions attended as a proportion of those offered).
3. Follow-up: numbers and proportion of participants completing follow-up and numbers not completing with reasons.
4. Independent rating of adherence to intervention manual/protocol (see above for details).

##### Data completeness

Data completeness for each questionnaire will be summarised, i.e. number of participants completing each questionnaire at each time point.

##### Unexpected adverse effects

Any unexpected adverse effects of participating in the trial will be recorded.

##### Acceptability of the intervention

Responses to a feedback questionnaire asking participants about their experience of the intervention will be recorded. The feedback questionnaire includes questions with rating scales and questions with written responses.

#### Secondary objective—collect data on clinical measures

Questionnaire measures of depression, anxiety and self-esteem as well as questionnaires and tasks that measure the proposed mechanisms of therapeutic change will be included. All measures are administered at each of the three assessment time points, except the ‘daily mood ratings and social connectedness’ and the ‘memory recall task’ which are only administered at pre-intervention and post-intervention time points.

1. Mood and Feelings Questionnaire (MFQ). The MFQ [59] is a self-report measure of depression. The long version of the MFQ (33 items rated on a 3-point Likert scale from 0 to 2) will be used at each of the three assessment time points and is the primary clinical measure for this trial. Scores range from 0 to 66 with higher score indicating more symptoms of depression. The MFQ has been used extensively in research and community settings; it is recommended by the National Institute of Clinical Excellence [57] and is routinely used in Child and Adolescent Mental Health services in the UK. The MFQ has been shown to be reliable and valid with scores of over 29 indicating a likely current major depressive episode and scores of over 20 indicating the presence of ‘any mood disorder’ [46]. For the screening stage, the four risk items will be removed from the MFQ due to ethical considerations in mass testing conditions. The MFQ at the screening stage will therefore consist of 29 items and a clinical cut-off score of 17 will be used at screening to reflect this. The shorter version (12 items) will also be administered at the beginning of each intervention session alongside a risk item to monitor any change in risk. The shorter version is preferred for intervention sessions given its quicker completion time.
2. Screen for Child Anxiety Related Disorders (SCARED). The SCARED [60] is a self-report measure of anxiety. The SCARED consists of 41 items, rated on a 3-point Likert scale from 0 to 2. Total scores on the measure range from 0 to 82 with higher scores indicating more anxiety. The SCARED also has five subscales: panic disorder or significant somatic symptoms, generalised anxiety disorder, separation anxiety, social anxiety disorder and significant school avoidance. The SCARED shows excellent internal consistency, test re-test reliability, and concurrent and discriminate validity [60, 61].
3. Revised Impact of Event Scale: child version (RIES-C). The 13-item self-report version of the RIES-C [62] will be administered to measure post-traumatic stress symptoms in reference to a negative event. Items are rated on a 4-point Likert scale (measured 0, 1, 3, 5) with higher scores indicating more symptoms of post-traumatic stress. There are also three subscales on the RIES-C: intrusion, avoidance and arousal. The RIES-C has been established as a good screen for PTSD diagnosis [62] with good internal reliability (Cronbach’s *α* = .80 [63]). The RIES-C also includes a single-item measure of intrusive image frequency.
4. Children’s Response Style Questionnaire (C-RSQ). The C-RSQ [64] measures cognitive responses to low mood using 25 items. It is a self-report measure with three subscales: ruminative responses, distractive responses and problem-solving responses. Young people are asked to rate how frequently they use certain responses to low mood. Items are rated on a 4-point Likert scale (measured 1 to 4) with higher scores indicating a greater tendency to engage in that response style. Previous studies have demonstrated good validity and good internal consistency [64, 65].
5. Rosenberg Self Esteem Scale (RSES). The RSES [66] is a ten-item self-report measure of self-worth. Scores range from 1 to 4 with higher scores indicating greater self-esteem. The RSES is widely used across the age range, and previous studies have demonstrated reliability, validity and good internal consistency in an adolescent sample [67–69].
6. Self-Concept Clarity scale (SCCS). The SCCS [70] measures a person’s confidence in being able to define themselves clearly. It is a self-report measure consisting of 12 items rated on a 5-point Likert scale (scored 1 to 5), with higher scores indicating greater self-concept clarity. Previous studies have demonstrated the validity and reliability of the SCCS [70], and it has been used in an adolescent population [71].
7. The Prospective Imagery Task (PIT). The adult version of the PIT [13, 17] has been adapted for use in young people. Participants are asked to read 14 scenarios, which includes 7 negative and 7 positive scenarios (e.g. ‘You will make good and lasting friendships’). Participants are asked to imagine each happening to them and then rate the vividness of the generated mental image on a 5-point scale (from 1 to 5). In addition to the adult version, participants will also be asked to specify how often they have had this image before on a 5-point scale (from 1 to 5). Higher scores indicate more vivid or frequent images of the future on the two scales.
8. Autobiographical Memory Task (AMT). The AMT [72] will be administered to measure memory specificity, following Williams and Broadbent procedure and coding scheme. Participants are asked to give a specific memory to ten cue words (five positive; five negative), presented in a randomised order orally and in writing (printed on index cards). Participants are given 60 s to respond to each cue word. Before beginning, participants are given the opportunity to practise two cue words (*beach* and *egg*) and are given feedback on their responses. Each memory will be coded as (1) ‘specific’ if the memory was of an event occurring at a particular place and time and lasting less than a day, (2) ‘general categorical’ if the memory was of a repeated event, (3) ‘general extended’ if the memory referred to an event that lasted longer than a day, (4) ‘semantic associate’ if the response was not a true autobiographical memory, or (5) ‘no response’. The AMT will be audio-recorded and the responses co-rated.
9. Daily mood ratings and social connectedness. Participants will be asked to rate ‘How *[emotion]* they feel’ on a Likert scale from 1 (not at all) to 9 (extremely) and to specify who they are with (family, friends, on my own, other: please specify). Mood ratings will be taken to eight emotions (happy, joyful, excited, energetic, sad, angry, nervous, and upset). Participants will be asked to install an app on their mobile phone and will be prompted to complete the questions once per day for 7 days pre-intervention and 7 days post-intervention. This app prompts them to answer the survey at the same time each day and also sends a reminder for them to complete it if they have not responded within an hour. If the app does not work for certain participants’ phones, then alternative methods will be used that best suit the participant (for example, text messages).
10. Memory recall task. A bespoke experimental task will measure participants’ emotional response to one positive autobiographical memory pre-intervention and a different positive autobiographical memory post-intervention. Participants will be asked to generate two positive autobiographical memories before attending the first assessment session and prior to randomisation. Emotional response will be measured using subjective ratings of mood (on the eight scales detailed above) as well as using a more objective psychophysiological measure, heart rate variability (HRV, recorded with Polar RCS800CX). This recall task was based on a recent experimental task using processing mode to alter positive emotional response [73].

#### Intervention development and development of training materials

Throughout the trial, qualitative descriptions of the treatment targets (e.g. negative images rescripted; positive future images generated) will be recorded. This will allow refinement of the intervention manual following trial completion and further development of training materials for teaching practitioners how to deliver the intervention.

#### Qualitative interviews

A small subset of trial participants will also be invited to take part in qualitative interviews following completion of their participation in the trial. These interviews will be conducted, analysed and reported separately from the main trial.

### Analysis plan

Primary feasibility data will be presented descriptively as outlined in the outcome measures section. Descriptive statistics will be reported for all other relevant outcomes at each time point by trial arm. This will include means (standard deviations) and medians (interquartile range) for normally and non-normally distributed variables, respectively. Frequencies and percentages will be used to summarise categorical data.

Additionally, for the secondary clinical and mechanistic outcomes, we will present between-group mean differences at the two follow-up time points in the form of standardised effect sizes with 95% confidence intervals, using both the intention-to-treat population (all participants randomised regardless of adherence to treatment) and the per protocol population (only participants adhering to treatment; defined as completing at least three sessions).

Flow through the trial will also be presented in a standard CONSORT diagram, including number approached to participate, number eligible/ineligible, number randomised, drop-outs before the end of treatment and numbers retained in the trial at 3-month follow-up.

#### Criteria for proceeding to a future trial

The decision of whether or not to proceed to a future definitive trial will be based on the feasibility and acceptability data. Primarily, to proceed, it will be important that recruitment for the current feasibility trial is achievable within a reasonable amount of time (2 years for full recruitment), retention rates are at least 80% at post-intervention and 70% at 3-months and an average acceptability of the ICBI intervention is rated as satisfactory or above. Not reaching these criteria does not necessarily indicate unfeasibility of a future definitive trial but may underline changes needed to the trial protocol, which will also be informed by qualitative findings regarding reasons for participation and any experiences of intervention which may impact upon retention or acceptability of the ICBI intervention. This would also be reviewed by the trial steering committee (TSC). Furthermore, any serious adverse events, serious adverse reactions or suspected unexpected serious adverse reactions that arise will be carefully reviewed by the TSC, and their advice will be followed on whether these would preclude proceeding to a definitive trial.

### Monitoring

#### Trial steering committee

A TSC has been formed. The TSC’s role is to provide oversight of trial progress and conduct (including adherence to protocol) and provide guidance for appropriate aspects of the trial. This includes monitoring and supervising the progress of the trial towards its interim and overall objectives and monitoring the rights, safety and well-being of the participants. Given the scope and nature of the trial, a data monitoring committee was not deemed necessary.

#### Criteria for discounting or modifying intervention for a given participant

The interventions will be discontinued for a given trial participant if adverse events are reported that are deemed to be associated with the intervention by the chief investigator (CI), supervisors and/or independent chair. The content of both the interventions will be tailored to the individual needs of the individual, whilst remaining consistent with the manuals.

#### Adverse events

Serious adverse events (SAE: events not necessarily caused by or related to the intervention), serious adverse reactions (SAR: events related to any duration of intervention administered to that person) and suspected unexpected serious adverse reaction (SUSAR: an adverse reaction where nature and severity of which is not consistent with the information known about the intervention in question in the view of the investigator) are defined as events that result in death, are life-threatening, require hospitalisation or prolongation of existing hospitalisation, result in persistent or significant disability or incapacity, or consist of a congenital anomaly or birth defect. All SARs and SUSARs will be reported immediately by the CI to the ethics committee within 15 days of the CI becoming aware of the event.

Adverse events (AE) (any untoward medical occurrence in a subject to whom an intervention has been administered including occurrences, which are not necessarily caused by or related to that therapy) will be monitored and recorded from randomisation to the final follow-up (3-month follow-up). As the trial will be conducted within a school setting with participants who are reporting symptoms of depression, possible AE that are likely in this population and will not be reported to the TSC are low-risk acts of self-harm (not requiring medical attention), e.g. *minor* scratching. Possible AE and serious adverse events (SAE) that are less likely but may still occur include high-risk acts of self-harm (requiring medical attention, but not medical hospital admission) and death by suicide. These will be reported to the independent chair of the TSC. Any adverse events that are considered by the CI, in consultation with the independent chair of the TSC, to be related to trial procedures in any way, or are unexpected, will be reported in timely fashion as stipulated above. These AE and SAE will be recorded and reported in full in the trial report and journal paper. In addition, risk and health service use will be assessed at each assessment time point, and a risk-monitoring question will be completed at each intervention session. The participant information sheet also contains information about who to contact if the participant feels that the study has harmed them in any way or if they wish to make a complaint about the conduct of the study.

##### Stopping guidelines

The trial may be prematurely discontinued by the Sponsor (The Director of Research Management and Innovation, King’s College London) or CI based on new safety information or for other reasons given by the ethics committee or TSC. If there is one adverse event, then the independent chair will be contacted; two adverse events and the TSC will be contacted; if there are three adverse events in a row then the trial would be paused to reflect and identify the problems, and the TSC would be contacted. The trial may also be prematurely discontinued due to lack of recruitment or upon advice from the TSC, who will advise on whether to continue or discontinue the study. If the study is prematurely discontinued, active participants will be informed. Next steps will be agreed upon in consultation with the independent chair; it is most likely that data collection would continue but that participants would no longer receive the intervention.

### Data management, modifications and publication

Participant data will be anonymised. Participants will be identified on the study database using a unique code and initials. All anonymised data will be stored on a password-protected computer and backed up regularly. All trial data will be handled, computerised and stored in line with the Data Protection Act, 1998 and archived in line with the Sponsor requirements. To assess data entry quality, the data will be checked using range checks and a small proportion of the entered data (10%) will be compared to the raw data by a member of the team blind to participant allocation. Further checks will be completed if necessary. Any modifications to the protocols (e.g. changes to eligibility criteria or assessments) will be reported to the relevant parties (e.g. ethics committee, TSC, ISRCTN) in a timely manner. It is intended that the results of the study will be reported and disseminated at the local level and more widely at national and international conferences and through publication in peer-reviewed scientific journals. The dataset will also be made publicly available following the completion of the trial.

### Service user involvement

Service user consultation has been integrated into the development of the intervention, the trial design and the trial protocol. Service user advisory groups were consulted during the development of the project and their recommendations incorporated. Two key examples of how service user consultation has influenced the design of the project were recommendations to have the inclusion criteria as young people with symptoms of depression (rather than a depression diagnosis) and delivering the intervention in schools (rather than in NHS clinics). Service users have been very positive about the aims of the trial, identifying a serious, unmet need for young people with depression, and a lack of clarity on what interventions could be offered. Two service user consultants have been recruited to provide consultation throughout the trial and to input on the TSC.

## Discussion

Here, we describe a feasibility RCT for a brief early intervention for adolescent depression. The intervention is anchored in cognitive science, developed in consultation with service users and designed to be delivered in secondary schools. The intervention aims to build positive future mental imagery, process distressing past negative imagery and improve specificity of autobiographical memories. These processes have been implicated in the development and maintenance of depression but have not previously been combined in a treatment package, such as this with an imagery focus.

A strength of the trial is that it can be readily scaled up into a definitive trial, should it be shown to be feasible, acceptable and safe. This includes collecting data on measures that assess mechanisms as these are important for a definitive trial to understand whether the active ingredients of the intervention are those proposed. Furthermore, a strength of the design is in the inclusion of an active control intervention: NDST. The control group in similar trials usually receive ‘no intervention’ or ‘waitlist’ condition (e.g. none of the indicated programmes reviewed by Calear and Christensen [8] received an active control). Given that change could be driven by factors related to non-specific aspects of therapy (e.g. speaking to an empathic therapist), it seems vital to include a control group that reflects what young people with depressive symptoms could receive in school from a school counsellor. However, the inclusion of such a group delivered by the first author also represents a weakness of the study. This control condition was chosen to reflect recommendations in NICE guidelines and ethical considerations of offering an appropriate intervention to young people with high symptoms of depression, and to ensure consistency within the control group. However, there is huge variability in the interventions delivered in schools to young people with high symptoms of depression as well as the availability of an intervention. Therefore, our control group perhaps reflects the upper end of what young people with high symptoms of depression are likely to receive as it is both a NICE recommended intervention and will be offered to all participants.

The design and methods proposed are commensurate with recommendations for feasibility studies [74], for example, being consistent with the guidelines provided by the National Institute for Health Research Trials and Studies (NETSCC). This feasibility study has been designed to enable us to test uncertainties that would arise when preparing for and undertaking a large-scale fully powered RCT of IBCI vs NDST. This trial will allow us to optimise both the intervention and the future trial design.

## Conclusion

The aim of this feasibility RCT is to determine whether an efficacy RCT is indicated and to aid the planning of such a trial. If the intervention is found to be feasible, acceptable and safe, then a future trial could test whether it is clinically and cost-effective. This could help improve the intervention options for young people with symptoms of depression, offering a novel early intervention for the early stages of adolescent depression.

## Abbreviations

AE: Adverse events
AMT: Autobiographical memory task
CBT: Cognitive behavioural therapy
CI: Chief investigator
C-RSQ: Children’s Response Style Questionnaire
ICBI: Imagery-based cognitive behavioural intervention
IMAGINE: Integrating Memories and Generating Images of New Experiences
KCTU: King’s Clinical Trials Unit
MFQ: Mood and Feelings Questionnaire
NDST: Non-directive supportive therapy
OGM: Overgeneral memory
PIT: Prospective Imagery Task
RCT: Randomised controlled trial
RIES-C: Revised Impact of Event Scale: child version
RSES: Rosenberg Self Esteem Scale
SAE: Serious adverse events
SAR: Serious adverse reactions
SCARED: Screen for Child Anxiety Related Disorders
SCCS: Self-Concept Clarity Scale
SUSAR: Suspected unexpected serious adverse reaction
TSC: Trial steering committee
UK: United Kingdom
VP: Victoria Pile
